# 611. Ethical Considerations in Treating Self- inflicted Burns: Impact on Healthcare Providers

**DOI:** 10.1093/jbcr/irag033.425

**Published:** 2026-04-06

**Authors:** Ashley E Honea, Monica L Gerrek, Marcie A Lambrix, Angharad Rees-Jones, Mary Rose E Rodda, Nathan H Brown, Shana Lennard, Tamara L Roberts, Claudia Islas, Erica Whetten, Karen J Richey, Kevin N Foster

**Affiliations:** Diane & Bruce Halle Arizona Burn Center - Valleywise Health, Phoenix, AZ; The MetroHealth System/Case Western Reserve University, Cleveland, OH; The MetroHealth System/Case Western Reserve University, Cleveland, OH; Santa Clara Valley Medical Center Regional Burn Center and Stanford University School of Medicine, San Jose, CA; Upstate University Hospital, Clark Burn Center, Syracuse, NY; University Medical Center, Louisiana State University Health Sciences Center, New Orleans, LA; LSU Health New Orleans/University Medical Center, New Orleans, LA; Suny Upstate Medical-Clark Burn Center, Blossvale, NY; Valleywise Health Medical Center, Phoenix, AZ; Valleywise Health Medical Center, Phoenix, AZ; Diane & Bruce Halle Arizona Burn Center - Valleywise Health, Phoenix, AZ; Diane & Bruce Halle Arizona Burn Center - Valleywise Health, Phoenix, AZ

## Abstract

**Introduction:**

The treatment and care of patients who have self-harmed or attempted suicide by burn present significant medical, psychological, ethical/legal, and emotional challenges for burn care teams. Despite their prevalence, there is limited research examining the unique considerations and impacts on healthcare providers caring for these patients. This study aims to fill this gap by investigating the challenges faced by burn healthcare providers when treating this population.

**Methods:**

A voluntary, anonymous survey was distributed to burn professionals at the 2025 American Burn Association Annual meeting and four burn centers. Participants accessed the survey via web link or QR code, with data stored in REDCap. Analysis included descriptive statistics and regression analysis of quantitative and qualitative participant feedback. Descriptive statistics summarized participant demographics, training needs, and support systems, and regression models assessed associations between case exposure and ethical considerations.

**Results:**

A total of 133 responses were collected, of which 106 (80%) were complete. Three primary training needs emerged that might help participants feel more confident treating these patients: understanding self-harm psychology (57%), legal/ethical considerations (54%), and emotional response management (46%). Most participants had 1-5 years of burn care experience, 32% disagreed that physical health should be prioritized over mental health, with 27% citing integration of mental health as the main care improvement. Exposure level was a meaningful predictor of ethical attitudes among healthcare staff with self-immolation linked to Justice, and self-injury to Respect for Autonomy. When asked about available support systems for staff emotional well-being, current staff support included counseling/resources (62%), debriefings/supervision (40%) and self-care/wellness programs (25%). Mental health service gaps included limited post-discharge follow-up (49%), inadequate inpatient resources (46%), and ethical/legal treatment complications (43%).

**Conclusions:**

This study reveals significant gaps in mental health support and training for burn professionals treating patients who have self-harmed, while demonstrating newer practitioners show greater awareness of the importance of mental health treatment in comprehensive care. The findings suggest an evolving paradigm toward integrated care models among emerging burn care professionals.

**Applicability of Research to Practice:**

This study highlights the need for burn care to prioritize mental health integration, develop targeted training programs to address self-harm psychology and ethical concerns, and establish comprehensive support systems for both patients and staff to improve care outcomes for this vulnerable population.

**Funding for the study:**

N/A.

